# Supplementary material for: Influenza A Virus Infection Reactivates Human Endogenous Retroviruses Associated with Modulation of Antiviral Immunity
Source: Viruses. 2022 Jul 21;14(7):1591. doi: 10.3390/v14071591 (PMC9320126; doi:10.3390/v14071591)
Supplement: Supplementary file 1 [file viruses-14-01591-s001.zip › MotifAnalysis.zip!/MotifOutput_PR8M/homerResults/motif11.similar.html]

motif11

## Information for motif11

C
T
A
G
A
C
G
T
C
T
G
A
A
C
G
T
A
G
T
C
A
C
G
T
C
G
T
A
A
C
T
G
A
G
T
C
A
C
G
T
T
C
G
A
G
T
A
C
  
Reverse Opposite:  

A
C
T
G
A
G
C
T
C
G
T
A
A
C
T
G
G
T
A
C
A
C
G
T
C
G
T
A
A
C
T
G
C
G
T
A
A
G
C
T
C
G
T
A
A
G
T
C
  

|  |  |
| --- | --- |
| p-value: | 1e-14 |
| log p-value: | -3.230e+01 |
| Information Content per bp: | 1.857 |
| Number of Target Sequences with motif | 8.0 |
| Percentage of Target Sequences with motif | 34.78% |
| Number of Background Sequences with motif | 158.4 |
| Percentage of Background Sequences with motif | 0.35% |
| Average Position of motif in Targets | 113.1 +/- 55.8bp |
| Average Position of motif in Background | 96.0 +/- 95.5bp |
| Strand Bias (log2 ratio + to - strand density) | 10.0 |
| Multiplicity (# of sites on avg that occur together) | 2.38 |
| Motif File: | file (matrix) reverse opposite |

### Similar de novo motifs found

|  |  |  |  |  |  |  |  |
| --- | --- | --- | --- | --- | --- | --- | --- |
| Rank | Match Score | Redundant Motif | P-value | log P-value | % of Targets | % of Background | Motif file |
| 1 | 0.742 | A C G T C T G A A C G T A C T G A C G T A G T C A C G T C G T A A C T G A G T C A C G T A G T C | 1e-13 | -32.001173 | 34.78% | 0.36% | motif file (matrix) |
| 2 | 0.923 | C T A G A C G T C T A G A C G T A G T C A C G T C G T A A C T G A G T C A C G T | 1e-13 | -31.622485 | 34.78% | 0.38% | motif file (matrix) |
| 3 | 0.730 | A C G T T C A G A C G T C T A G A C G T A G T C A C G T C G T A C T A G A G T C | 1e-12 | -29.280333 | 34.78% | 0.50% | motif file (matrix) |
| 4 | 0.843 | A C G T C T G A A C G T A G T C A C G T C G T A A C T G A G T C | 1e-11 | -25.381205 | 30.43% | 0.46% | motif file (matrix) |
| 5 | 0.791 | A C G T A C T G A C G T A G T C A C G T C G T A A C T G A G T C | 1e-9 | -22.055819 | 30.43% | 0.74% | motif file (matrix) |
